# The connection between nonlinear extension of Maxwell’s equations, blackbody spectrum, Lorentz force and quantum mechanics

**DOI:** 10.1038/s41598-025-34478-2

**Published:** 2026-01-19

**Authors:** Shiva Kumar

**Affiliations:** Electrical and Computer Engineering, ITBA-314, McMaster University, 1280 Main St. W., Hamilton, ON L8S 4K1 Canada

**Keywords:** Mathematics and computing, Physics

## Abstract

We propose that the electromagnetic field in vacuum is governed by a Lorentz-invariant nonlinear extension of Maxwell’s equations. Numerical simulations of this model show that cavity modes interact via four-wave mixing, leading to an equilibrium spectrum that closely matches the blackbody spectrum. A fit to the Planck distribution suggests that the vacuum’s nonlinear coefficient is inversely proportional to Planck’s constant, linking this nonlinear effect to energy quantization. The equations also admit dark soliton solutions whose mean energy is proportional to $\hbar \omega$, directly connecting field structure to the quantum energy–frequency relation. Furthermore, the nonlinear formulation naturally yields the Lorentz force and provides an alternative explanation for phenomena such as the photoelectric effect, usually requiring quantum postulates. Our results motivate experimental tests of vacuum nonlinearities and offer a new perspective on unifying classical and quantum descriptions of light–matter interaction.

## Introduction

In Maxwell’s theory, field equations do not provide the equations of motion for charged particles; equations of motion are given separately by Lorentz force equations. In contrast, in the case of Einstein’s field equations, they describe not only the evolution of the gravitational field due to mass points, but also provide the equations of motion for the mass points. Bergmann attributed this to the nonlinear nature of Einstein’s equations^1^. Inspired by this fact, we investigate whether introducing a nonlinear term into Maxwell’s equations could unify EM field dynamics with the Lorentz force equations.

Einstein postulated that the energy of a photon is proportional to the frequency of radiation^2^. Later, $E = \hbar \omega$ became one of the foundational postulates of quantum mechanics and quantum field theory. However, this postulate is not derived from first principles. Nonlinear optics is rich with examples where wave energy and frequency become coupled through soliton formation. In linear EM theory, the amplitude and frequency of a wave packet are independent degrees of freedom. But certain nonlinear waves and solitons naturally link these quantities. Motivated by this, we explore a Lorentz-invariant nonlinear generalization of Maxwell’s equations that admits dark soliton solutions whose energy is proportional to $\hbar \omega$, mirroring the quantum energy–frequency relation for photons.

In 1925, de Broglie attempted to develop a double solution theory in which the wave is unified with the particle in the 4D spacetime and not in the configuration space^3–6^. In this theory, the particle is a singularity riding on a physical pilot wave, and these objects were nonlinearly coupled, quite similar to solitary waves. However, soliton theory was not advanced then, and de Broglie abandoned this theory in 1928 due to its mathematical complexity^7^. Quantum electrodynamics (QED) predicts nonlinear optical effects in vacuum due to the presence of virtual electron-positron pairs^8–10^. Optical Kerr effect in vacuum is previously investigated by Aleksandrov and Moskalev^11^ and by Robertson^12^ using QED. Robertson has found that the optimal value of nonlinear index of vacuum derived from the Euler-Heisenberg model is 1.555$\times 10^{-33}$ cm$^2$/W for a linearly polarized pump as seen by the counter-propagating orthogonally polarized probe^12^. In this paper, it is not assumed that the nonlinear optical effects in vacuum are due to QED; instead, it is postulated that the evolution of electromagnetic (EM) field in vacuum is described by the Lorentz invariant nonlinear extension of Maxwell’s equations. For the weak EM fields, they reduce to the conventional linear Maxwell’s equations. Numerical simulations of the nonlinear equations show that modes in a closed cavity exchange energy through four-wave mixing and settle into an equilibrium spectrum resembling the blackbody radiation curve. Fitting this spectrum to Planck’s distribution reveals a direct connection between the nonlinear coefficient of vacuum and Planck’s constant.

## Nonlinear extension of Maxwell’s equations

In this paper, it is postulated that EM propagation in a vacuum is described by a nonlinear extension of Maxwell’s equations,

1 $$\begin{aligned} \nabla ^2 A^\mu -\frac{1}{c^2} \frac{\partial ^2 A^\mu }{\partial t^2} = \kappa A^2 A^{\mu }, \end{aligned}$$

where $A^2 = g_{\alpha \beta }A^{\alpha }A^{\beta }$, $A^\mu$ is the vector potential, $g_{\alpha \beta }$ is Minkowski tensor, $\kappa$ is the nonlinear coefficient of vacuum, assumed to be sufficiently small so that EM propagation in a vacuum is essentially linear for moderate EM field intensities. The vector potentials satisfy Lorentz conditions,

2 $$\begin{aligned} \partial _\mu A^\mu =0. \end{aligned}$$

To be consistent with the theory of relativity, Maxwell’s equations should be Lorentz Invariant. It can be shown that nonlinear Maxwell’s equations are Lorentz invariant by considering an observer moving at a speed *v* in a frame $K'$ relative to an observer in a frame *K* (SI).

For a one-dimensional EM plane wave with electric field $E_x$, magnetic field $H_y$, and the wave is propagating in *z* direction, (1) leads to [Methods]

3 $$\begin{aligned} n = 1 + n_2 \mathcal {E}_{EM}, \end{aligned}$$

where *n* is refractive index, $n_2$ is the effective Kerr coefficient, and $\mathcal {E}_{EM}$ is EM energy density. (3) implies the vacuum refractive index depends weakly on the local EM energy density, analogous to the Kerr effect in nonlinear optics (see Methods for a thought experiment illustrating this concept). We note that the vacuum filled with intense EM radiation is not the true vacuum, and its refractive index differs from unity as given by (3), which is quite similar to the Gladstone-Dale equation^13^. For an ideal gas with refractive index $n \approx 1$, the change in refractive index is related to energy density $\rho$ using Gladstone-Dale equation

4 $$\begin{aligned} n = 1 + K \rho , \end{aligned}$$

where *K* is the Gladstone-Dale constant. To measure the small refractive index change due to vacuum nonlinearity given by (3), we need an experimental setup with a near-vacuum condition in an almost perfectly reflecting cavity filled with intense EM radiation.

## Spectrum at equilibrium

Consider the electromagnetic propagation and interaction in a cavity that is a cube of length *L*. In the absence of the nonlinear term in (1), the cavity admits transverse electric (TE) modes and transverse magnetic (TM) modes^14^.

In the absence of the nonlinear term, these modes do not interact. For example, if the modes are excited with specific weights initially, the mode weights do not change as a function of time. Hence, the spectrum remains time-invariant. However, in the presence of the nonlinear term, these modes could interact and exchange energy, leading to a spectrum significantly different from the initial spectrum.

(1) is solved numerically using the fourth-order Runge-Kutta technique (SI). A few of the TE modes of the cavity are excited initially. Due to the nonlinear term on the right-hand side of (1), these modes interact nonlinearly and exchange energy due to four-wave mixing (FWM). Also, their nonlinear interaction leads to new frequency components. The spectrum gradually widens due to FWM, and eventually, some kind of equilibrium state is achieved after which the spectrum becomes nearly invariant. We refer to this as the equilibrium spectrum.

**Fig. 1:**
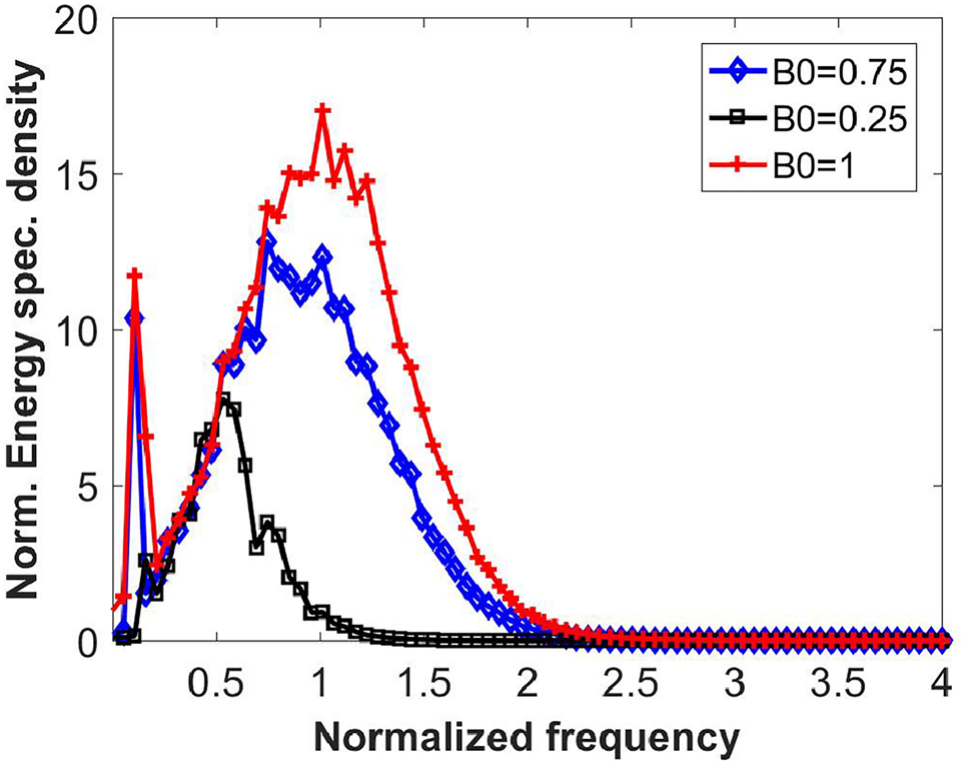
Equilibrium spectrum after $t'$=3600. $\kappa '$ = 0.1 and *L*=10. See the SI for the parameters used in the simulations.

Fig. 1 shows the equilibrium spectrum. Here, $B_0$ represents the initial weights of TE modes excited. As $B_0$ increases, the nonlinear interaction between the modes due to FWM becomes stronger, generating new frequency components which widens the spectrum. These curves resemble the blackbody spectrum except for the peaks near the zero-frequency (See SI for an explanation).

**Fig. 2:**
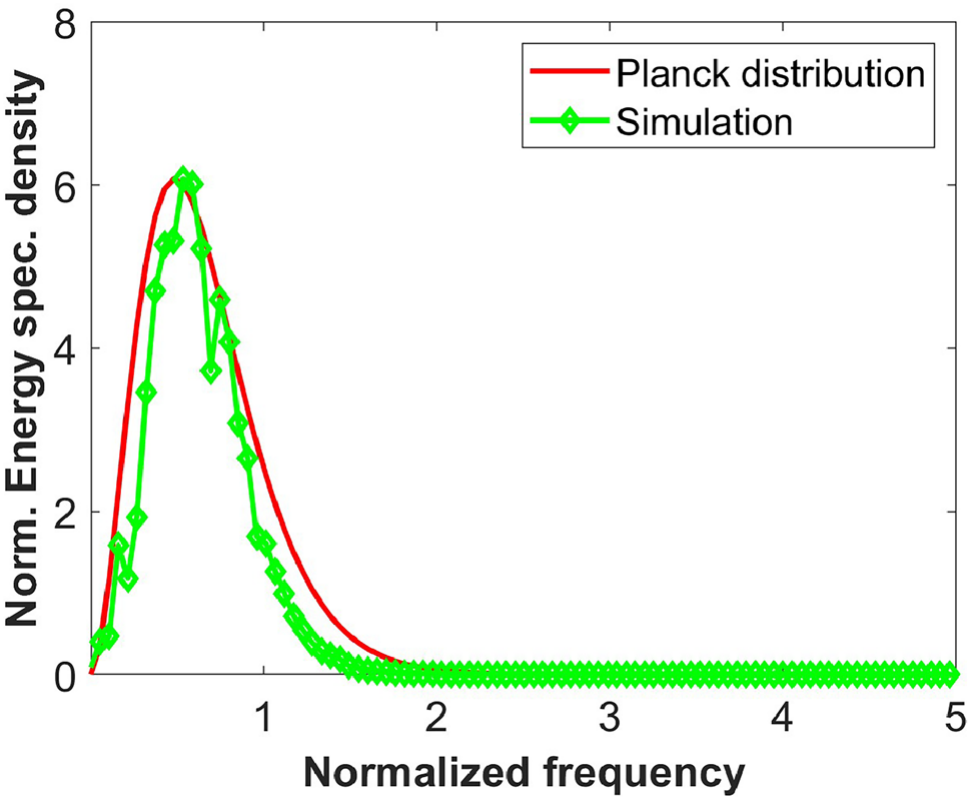
Equilibrium spectrum. The parameters are the same as Fig. 1 with $B_0$ = 0.25. The red curve shows the Planck distribution with *a* = 833.88, and *b*=5.8 (see Methods), while the green curve shows the results of the numerical simulation of (1).

When $B_0$ is small, the peak near the zero-frequency is small, and in that case, to see the resemblance between the blackbody spectrum and simulated spectrum of Fig. 1, a curve fitting to the equilibrium spectrum using the Planck distribution is carried out. As can be seen from Fig. 2, there is a good match between the Planck distribution and the numerical simulation of (1). After further processing [Methods], we find that the nonlinear coefficient satisfies:

5 $$\begin{aligned} \kappa \approx \frac{1}{\hbar c}, \end{aligned}$$

establishing a direct link between the nonlinear extension of Maxwell’s equations and Planck’s constant.

Planck was aware that if a perfectly reflecting boundary is used and if the radiation evolves according to Maxwell’s equations (linear) in the cavity, it would not lead to a blackbody spectrum (simply because the linear modes do not interact with each other, leading to new frequency components)^15^ (See SI). He expected to have an arbitrarily small amount of matter, such as a molecule of carbon, in the cavity, to obtain the blackbody spectrum. The high-intensity EM radiation introduced through the nonlinear term in Eq. (1) may be considered as a substitute for Planck’s carbon molecule.

## Dark soliton solutions

Let us consider the case ${\textbf {A}} = A^{\phi } \boldsymbol{\phi }$ and the rest of the vector EM potential components are zero. We look for a spherically symmetric dark soliton solution. Let

6 $$\begin{aligned} A^{\phi } = \frac{1}{r^2} [ \psi (r) \exp (i\omega t) + c.c. ]. \end{aligned}$$

Substituting (6) in (1) [Methods], we find

7 $$\begin{aligned} \frac{d^2 \psi }{dr^2} + \left[ \frac{\omega ^2}{c^2} - \frac{2 + 2\kappa \psi ^2(r)/3}{r^2} \right] \psi = 0. \end{aligned}$$

**Fig. 3:**
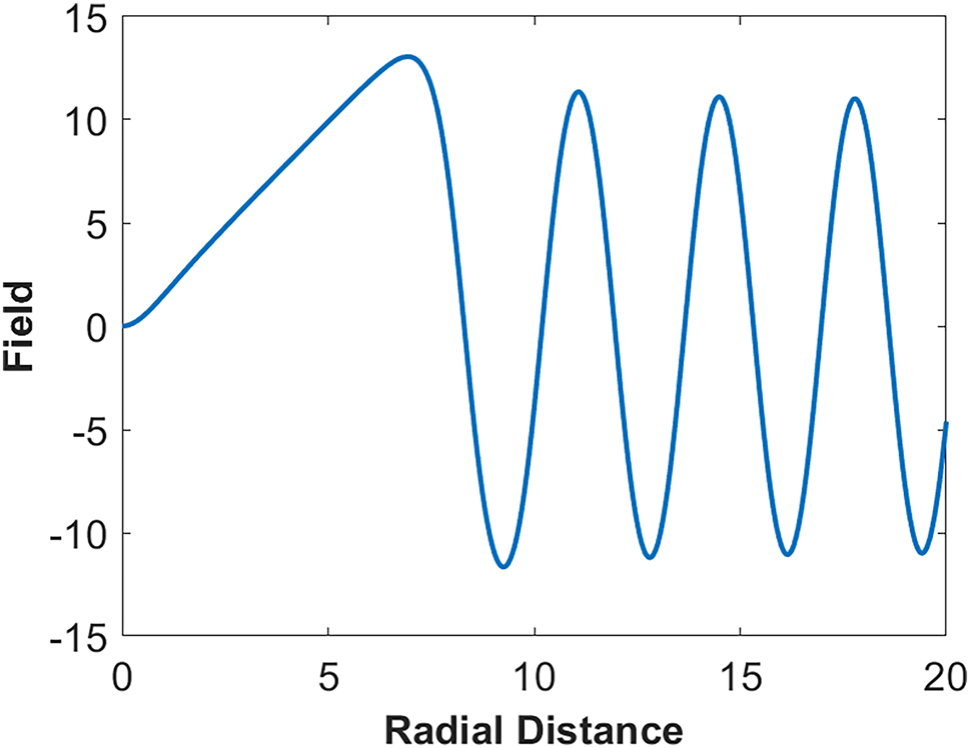
Field distribution $\psi$ as a function of the radial distance *r*. Normalized parameters are used. $\kappa _{eff}$ = 1, $\omega = 2$, and $c=1$. Initial conditions: at $r=10^{-4}$, $\psi = 3.0515\times 10^{-8}$, and $d \psi /(dr) = 3.02\times 10^{-4}.$

(7) is solved numerically, and Fig. 3 shows the field distribution $\psi$ as a function of the radial distance *r*. From (7), we find that as $r \rightarrow \infty$, $\psi \rightarrow \sin (\omega r/c + c_0)$, where $c_0$ is a constant. In Fig. 3, we see that the field has a ’hole’ at the center, and as *r* becomes larger, the field becomes a sinusoid.

The energy of a dark soliton is unbounded since it becomes a sinusoid as $r \rightarrow \infty$. Typically, the energy of a dark soliton is defined as the difference between the total energy and the background energy. The energy of a dark soliton is found to be proportional to its mean frequency [Methods]

8 $$\begin{aligned} E_{em}' = M \hbar \omega , \end{aligned}$$

where *M* is a constant. For this ground state dark soliton solution, the field is independent of $\theta$. However, for the higher order states, the field depends on $\theta$, and it can be shown that the energy of a higher order state is also proportional to $\hbar \omega$, and *M* for a higher order state is higher than that of the ground state dark soliton. The existence of fundamental and higher-order dark solitons may be verified in a laboratory setup, provided that near-vacuum conditions can be achieved in a spherical cavity.

### Dark soliton excitation

In the context of optical solitons, although the existence of dark solitons was theoretically predicted in 1973^16^, it took 14 years to realize it experimentally^17,18^. Since the ideal dark soliton has a continuous wave (CW) background that extends to infinity, it was harder to realize it experimentally. If the width of the CW background region is sufficiently longer than the “hole” region, an ideal dark soliton can be approximated by a dark pulse with finite, but long background. In our case, if we have near-vacuum conditions inside a spherical cavity with its radius much longer than the width of the “hole” region, it may be possible to approximate the ideal dark soliton. If we launch a CW field into the cavity, with its amplitude *S* corresponding to the CW background of the dark soliton and its energy close to that of a dark soliton, it is likely to excite a fundamental dark soliton of the form shown in Fig. 3. As the amplitude increases, higher-order solitons can be excited.

### Interaction of dark solitons

Let

9 $$\begin{aligned} (A^\phi )_j = \frac{1}{r^2}[\psi _j(r) \exp (i \omega _j t) + c.c.], \hspace{0.2in} j =1,2 \end{aligned}$$

be two dark solitons with frequencies $\omega _1$ and $\omega _2$. Let

10 $$\begin{aligned} A^\phi = \sum _{j=1}^2 (A_\phi )_j. \end{aligned}$$

Substituting (10) in (1), we find that the nonlinear interaction between two dark solitons leads to new frequency components at $\Omega _1 = 2\omega _1-\omega _2$ and $\Omega _2 = 2\omega _2-\omega _1$, due to four-wave mixing (FWM) (see SI). Let $\omega _1 = m \Omega$ and $\omega _2 = (m+1) \Omega$ where $\Omega$ is a small fixed frequency. In this case, we find $\Omega _1 = (m-1)\Omega$, and $\Omega _2 = (m+2)\Omega$. Thus, two dark solitons of frequencies $m \Omega$ and $(m+1) \Omega$ generate FWM components of frequencies $(m-1)\Omega$ and $(m+2)\Omega$. The nonlinear interaction between the frequency components $(m-1) \Omega$, $m \Omega$, and $(m+1) \Omega$ leads to a FWM component at $(m-2)\Omega$. The nonlinear interaction between the frequency components $m \Omega$, $(m+1) \Omega$, and $(m+2) \Omega$ leads to a FWM component at $(m+3)\Omega$, and so on. Ultimately, an equilibrium state will be reached such that there would no longer be energy exchange due to nonlinear interaction, and this equilibrium spectrum is nothing but the blackbody spectrum.

## Coulomb force and Lorentz Force

The time component of (1) can be written as

11 $$\begin{aligned} \nabla ^2 A^0 -\frac{1}{c^2} \frac{\partial ^2 A^0}{\partial t^2} = -\frac{\rho _{p}}{\sqrt{\epsilon _0}} , \end{aligned}$$

where $\rho _{p}$ is the charge density of pseudo-charges, $\rho _{p} = -\sqrt{\epsilon _0}\kappa A^2 A^0$. Let the pseudo-charge be

12 $$\begin{aligned} q_{p} = \int \rho _{p} dV. \end{aligned}$$

The interaction between real charges may not be described by (1). However, the interaction between two pseudo-charges is quite similar to that between the real charges, and it can be described by (1) as shown in the following subsections. Due to self-similarity, the spherically symmetric and time-independent solution of (11) can be written as

13 $$\begin{aligned} A^0 = \frac{\eta }{\sqrt{\kappa }} f(\eta r), \end{aligned}$$

with $f(x) \rightarrow 1/x$ as $x \rightarrow \infty$. Using (13), (12), and Gauss’s divergence theorem [Methods], we find

14 $$\begin{aligned} q_{p}^2 = 4\pi \epsilon _0 \hbar c K, \end{aligned}$$

where *K* is a dimensionless constant. For a real electric charge, *K* is equal to the fine structure constant, $\alpha \approx 1/137$.

### Coulomb force

The Coulomb interaction between pseudo-charges can be described by the time component ($\mu$=0) of $A^\mu$ while other components ($\mu$=1,2,3) describe the spin of the pseudo-charge. In this subsection, we focus only on the time component $A^0$ and ignore other components. Let

15 $$\begin{aligned} A^0 ({\textbf {r}},t) = \frac{1}{2}[\rho ({\textbf {r}},t) \exp (-i\omega t) + c.c.], \end{aligned}$$

where $\rho ({\textbf {r}},t)$ is the slowly varying envelope. Although $A^0$ can have a zero-frequency component, it is ignored in this section. Substituting (15) in (11), and making the slowly varying envelope approximation, we obtain

16 $$\begin{aligned} \frac{2i\omega }{c^2} \frac{\partial \rho }{\partial t} + \nabla ^2 \rho = -\frac{3 \kappa }{4} |\rho |^2 \rho . \end{aligned}$$

Let there be two identical pseudo-charges centered at $z_0/2 \vec {z}$ and $-z_0/2 \vec {z}$ with the separation of $z_0$ between them. Let

17 $$\begin{aligned} \rho ({\textbf {r}},t) = \rho _1({\textbf {r}},t) + \rho _2({\textbf {r}},t), \end{aligned}$$

where $\rho _1$ and $\rho _2$ correspond to the charge distributions of pseudo-charge 1 and 2, respectively. Using (17) in (16), we find the rate of change of momentum of pseudo-charge 1 due to its interaction with pseudo-charge 2 as (SI)

18 $$\begin{aligned} \frac{d \langle p_z \rangle }{dt} = -\frac{3 \kappa }{4}Re[ \langle \rho _1 |\rho _1^\star \tilde{E}_2|\rho _1 \rangle + 2\langle \rho _1 |\rho _2 \tilde{E}_1^\star |\rho _1 \rangle ], \end{aligned}$$

where $\langle p_z \rangle$ is the mean of the *z*-component of the momentum of pseudo-charge 1, and $\tilde{E}_j$ is the slowly varying envelope of electric field intensity due to pseudo-charge *j*. The first term on the right-hand side can be identified with the Coulomb force. However, the second term seems to have no analog in the context of interaction between the real charges, although it has some resemblance to radiative reaction^19^. Nevertheless, in the limit of the charge distribution becoming point-like, the second term in (18) will be significantly smaller than the first term.

### Lorentz force

Consider a single pseudo-charge stationary in a reference frame *K*. Let the vector potential due to this charge be $A^\mu$. Consider an observer moving at the speed $v_x$ in the *x*-direction. Let the reference frame of the observer be $K'$ with primed coordinates. Using the Lorentz transformation, (1) can be written in the frame of $K'$ with vector potentials $A'^\mu$. Let $\vec {A'}_{ext} = (A^x)'_{ext} \vec {x}$ be the vector potential due to an external magnetic field that is stationary in $K'$ frame. Taking into account the Lorentz invariance of the nonlinear Maxwell’s equations, (1), we find the rate of change of momentum of the pseudo-charge in the $K'$ frame as (SI)

19 $$\begin{aligned} \frac{d \langle p_z \rangle }{dt} =-\frac{\kappa }{2c} Re[ \langle \phi ^0 |(\phi ^0)^\star v_x (\tilde{B}^y_{ext})|\phi ^0 \rangle + 2c\langle \phi ^0 | (\tilde{B}^{y'})^\star \phi ^x_{ext}|\phi ^0 \rangle ]. \end{aligned}$$

where $\phi ^0$ is the slowly varying envelope of $(A^0)'$, $\tilde{B}^y_{ext}$ and $\tilde{B}^{y'}$ are the slowly varying envelopes of the external magnetic field and that due to the pseudo-charge, respectively. The first term on the right-hand side of (19) can be identified as a Lorentz force due to the magnetic field $\tilde{B}^y_{ext}$ on the pseudo-charge moving at a speed of $-v_x$ with respect to the external magnetic field. The second term is likely to be associated with the radiative reaction.

Finally, we note that if we define the effective mass of the pseudo-charge as $m_{eff} = \hbar \omega /c^2$, and using $\kappa = 1/(c\hbar )$, (16) can be cast as the exact Schrodinger equation,

20 $$\begin{aligned} i\hbar \frac{\partial \rho }{\partial t} + \frac{\hbar ^2}{2m_{eff}} \nabla ^2 \rho = V \rho , \end{aligned}$$

where $$V = -\frac{3c}{8 \omega } |\rho |^2$$.

## Photoelectric effect

If the frequency of a light wave exceeds a certain threshold frequency, electrons are ejected from a material surface. However, linear Maxwell’s equations combined with the Lorentz force cannot explain this frequency dependency. To explain the threshold frequency, Einstein postulated that a lightwave of frequency $\omega$ is made up of photons of energy $\hbar \omega$. In this paper, an alternative explanation for the frequency dependency based on the FWM phase matching is provided. We assume that the stationary states of the pseudo-charge are of the form

21 $$\begin{aligned} A^0_j = \frac{1}{2}[\rho _{0j}(\textbf{r}) + B_j \rho _j(\textbf{r})\exp (i\omega _j t) + c.c.], \hspace{0.2in} j=1,2,3\ldots \end{aligned}$$

We consider the interaction between two states with frequencies $\omega _h$ and $\omega _l$ with $\omega _h> \omega _l$ mediated through an external electromagnetic field,

22 $$\begin{aligned} A^0_{ext} = \frac{1}{2} [\rho _{ext}(\textbf{r},t) \exp (i\omega _{ext} t) + c.c.], \end{aligned}$$

where $\omega _{ext}$ and $\rho _{ext}$ are its frequency and slowly varying envelope, respectively. The total time component of the vector potential is $A^0= A^0_l + A^0_h + A^0_{ext}$. Substituting this into (11) and simplifying, we find (SI)

23 $$\begin{aligned} i\hbar \frac{dB_l}{dt}&= B_h \Gamma _l \exp (i\Delta \omega t), \nonumber \\ i\hbar \frac{dB_h}{dt}&= B_l \Gamma _h \exp (-i\Delta \omega t), \end{aligned}$$

24 $$\begin{aligned} 2i \frac{\omega _{ext}}{c^2} \frac{\partial \rho _{ext}}{\partial t} - \nabla ^2\rho _{ext} = \frac{3 \kappa B_h B_l^{\star } \rho _h \rho _l^{\star } (\rho _{0r}^r + \rho _{0l}^r)\exp (i\Delta \omega t)}{2}, \end{aligned}$$

where $\Delta \omega = \omega _h - \omega _l - \omega _{ext}$ is the FWM phase mismatch. When $\Delta \omega = 0$, the efficiency of the FWM process is maximum. If the pseudo-charge is initially in the state of frequency $\omega _l$ and after interacting with the external EM field, it makes a transition to the state of frequency $\omega _h$ provided the FWM phase matching condition, $\Delta \omega = 0$ is satisfied. Alternatively, if we associate energies, $E_h = \hbar \omega _h$, $E_l = \hbar \omega _l$, and $E_{ext} = \hbar \omega _{ext}$, the FWM phase matching condition becomes, $E_h = E_l + E_{ext}$. Einstein explained this effect as an electron of energy $E_l$ absorbing a photon of energy $\hbar \omega _{ext}$ and making the transition to the state with energy $E_h$.

If the pseudo-charge is initially in the state of higher frequency, $\omega _h$, it could make a transition to the state of lower frequency $\omega _l$ if the FWM phase matching condition, $\Delta \omega = 0$ satisfied. In this case, on solving (24), we find that the external EM field grows exponentially, which can be interpreted as stimulated emission. Stimulated Compton scattering in vacuum, occurring due to the interaction between a free electron and photons of different frequencies^20^, can be explained as the cascaded FWM. In fact, any type of interaction between an electron and EM radiation can be explained using the model of (1), provided the electron is replaced with the pseudo-charge.

## Conclusions

We have shown that a Lorentz-invariant nonlinear extension of Maxwell’s equations yields rich dynamics that connect nonlinear field theory with key features of quantum mechanics. The model produces a cavity equilibrium spectrum matching the blackbody distribution, links the nonlinear coefficient to Planck’s constant, generates dark soliton solutions with energy proportional to $\hbar \omega$, and recovers the Lorentz force and photoelectric effect through nonlinear interactions. The proposed model is in good agreement with quantum mechanics, and it provides more insight into the fundamental postulates of quantum mechanics. These results suggest that quantum behavior may emerge from the nonlinear extension of Maxwell’s equations, motivating further theoretical work and possible experimental probes of vacuum nonlinearities. The open question is if the nonlinear waves described by (1) are deterministic or probabilistic.

## Methods

### A. Dependence of refractive index on EM energy density

Consider a one-dimensional electromagnetic (EM) plane wave with electric field $E_x$, magnetic field $H_y$, and the wave is propagating in *z* direction. For this case, we have

25 $$\begin{aligned} E_x = -\frac{1}{c}\frac{\partial A^x}{\partial t}, \end{aligned}$$

26 $$\begin{aligned} B_y = \frac{\partial A^x}{\partial z}, \end{aligned}$$

and $A^y = A^z = A^0 = 0$. Now (1) becomes

27 $$\begin{aligned} \frac{\partial ^2 A^x}{\partial z^2} - \frac{1}{c^2}\frac{\partial ^2 A^x}{\partial t^2} = \kappa (A^x)^3. \end{aligned}$$

Let

28 $$\begin{aligned} A^x = \frac{1}{2} \left[ A_{x0} \exp [i(\omega t - k z)] + c.c. \right] . \end{aligned}$$

Substituting (28) in (27) and ignoring the higher frequency components $\exp [\pm i3(\omega t - kz)]$, we find

29 $$\begin{aligned} \frac{\omega ^2}{c^2} - k^2 = \frac{3}{4}\kappa |A_{x0}|^2 . \end{aligned}$$

Using (28) in (25) and (26), we have

30 $$\begin{aligned} E_x = \frac{-\omega }{2c} \left[ i A_{x0} \exp [i(\omega t - k z)] + c.c. \right] , \end{aligned}$$

31 $$\begin{aligned} B_y = \frac{-k}{2} \left[ i A_{x0} \exp [i(\omega t - k z)] + c.c. \right] . \end{aligned}$$

Using (30) and (31), the mean EM energy density is

32 $$\begin{aligned} \mathcal {E}_{EM}&= \frac{1}{2} [ \langle E_x^2 \rangle + \langle B_y^2 \rangle ], \nonumber \\&= \frac{1}{4} \left( \frac{\omega ^2}{c^2} + k^2\right) |A_{x0}|^2. \end{aligned}$$

Using (32), (29) is rewritten as

33 $$\begin{aligned} \frac{\omega ^4}{c^4} - k^4 = 3 \kappa \mathcal {E}_{EM} . \end{aligned}$$

Defining the phase velocity as $v_{ph} = \omega /k$ and simplifying (33), we find

34 $$\begin{aligned} v_{ph}&= c\left( 1 + \frac{3\kappa \mathcal {E}_{EM}}{k^4} \right) ^{1/4}, \nonumber \\&\approx c\left( 1 + \frac{3\kappa \mathcal {E}_{EM}}{4k^4}\right) . \end{aligned}$$

Refractive index *n* is defined as $c/v_{ph}$. From (34), we have

35 $$\begin{aligned} n \approx 1 + n_2 \mathcal {E}_{EM}, \end{aligned}$$

where $n_2$ is the effective Kerr coefficient

36 $$\begin{aligned} n_2 = -\frac{3\kappa }{4k^4}. \end{aligned}$$

The rationale for the second term on the right-hand side of (35) that is proportional to the energy density can also be explained from the following thought experiment: imagine a perfectly reflecting hollow sphere surrounding an electron and a positron that are yet to collide. If a weak probe of an electromagnetic wave is incident on this system, it would find the refractive index to be unity everywhere inside the sphere except at the locations of the electron and the positron. The result of the collision is the annihilation of the electron and the positron, and the creation of an electromagnetic wave. If the walls of the sphere are perfectly reflecting, electromagnetic energy would be confined to the sphere. In this case, it is unreasonable to expect the weak electromagnetic wave to find the refractive index to be unity everywhere because it was not unity everywhere before the collision. Therefore, we expect that the refractive index to deviate slightly from unity everywhere within the sphere, with the deviation of the refractive index being proportional to the local energy density of electromagnetic wave created by the collision. Before the collision, the deviation of refractive index from unity is mostly confined to the locations of electron and positron. The distinction between the probe EM wave and that created by collision is artificial, and so, the deviation of the refractive index from unity for an EM field should be proportional to the local EM energy density due to its own field as well as other EM fields. This is the origin of the nonlinear effect in (1).

### B. Conservation of pseudo-charge

Let

37 $$\begin{aligned} \textbf{B} = \nabla \times \textbf{A}, \nonumber \\ \textbf{A} = -\nabla \phi -\frac{1}{c} \frac{\partial \textbf{A}}{\partial t}. \end{aligned}$$

We define the 4-vector potential as $\overrightarrow{\mathbf{{A}}}=(A^0,A^1,A^2,A^3)$, and $A^0 = \phi$. Using (37) and Lorentz condition (2), (1) can be recast as

38 $$\begin{aligned} \nabla . \textbf{E}&= \frac{\rho _p}{\sqrt{\epsilon _0}}, \end{aligned}$$

39 $$\begin{aligned} \nabla . \textbf{B}&= 0, \end{aligned}$$

40 $$\begin{aligned} \nabla \times \textbf{E}&= -\frac{1}{c}\frac{\partial \textbf{B}}{\partial t},\end{aligned}$$

41 $$\begin{aligned} \nabla \times \textbf{B}&= \sqrt{\mu _0} \textbf{J} + \frac{1}{c} \frac{\partial \textbf{E}}{\partial t}, \end{aligned}$$

where

42 $$\begin{aligned} \rho _p = -\sqrt{\epsilon _0}\kappa A^2 \phi , \nonumber \\ \textbf{J} = -\frac{\kappa A^2 \textbf{A}}{\sqrt{\mu _0}} ,\nonumber \\ A^2 = A^\mu A_\mu . \end{aligned}$$

Taking the divergence of (41) and noting that the divergence of the curl of a vector is zero, we find

43 $$\begin{aligned} \nabla . (\nabla \times \textbf{B}) = \sqrt{\mu _0} \nabla .\textbf{J} + \frac{1}{c}\frac{\partial }{\partial t} (\nabla .\textbf{E})=0. \end{aligned}$$

Using (38) in (43), we find

44 $$\begin{aligned} \nabla .\textbf{J} = - \frac{\partial \rho _p}{\partial t} . \end{aligned}$$

We define the 4-current as $\overrightarrow{\textbf{J}}=(c\rho _p,J^1,J^2,J^3)$ so that

45 $$\begin{aligned} J^\mu = -\frac{\kappa A^2 A^\mu }{\sqrt{\mu _0}}. \end{aligned}$$

Using the 4-current $\overrightarrow{\textbf{J}}$, pseudo-charge continuity equation (44) can be rewritten as

46 $$\begin{aligned} \partial _\mu J^\mu = 0. \end{aligned}$$

### C. Lagrangian density for the nonlinear Maxwell’s equations

Let

47 $$\begin{aligned} F_{\mu \nu } = \partial _\mu A_\nu - \partial _\nu A_\mu , \end{aligned}$$

Using (47), inhomogeneous Maxwell’s equations ((38) and (41)) can be rewritten as

48 $$\begin{aligned} \partial _\mu F^{\mu \nu } = \sqrt{\mu _0} J^\mu , \end{aligned}$$

The Lagrangian density associated with nonlinear Maxwell’s equations is

49 $$\begin{aligned} \mathcal {L} = \mathcal {L}_{free} + \mathcal {L}_{int}, \end{aligned}$$

where

50 $$\begin{aligned} \mathcal {L}_{free} = -\frac{1}{4}F_{\mu \nu }F^{\mu \nu }, \end{aligned}$$

51 $$\begin{aligned} \mathcal {L}_{int} = \frac{\kappa }{4} A^4 . \end{aligned}$$

The Euler-Lagrangian field equations are

52 $$\begin{aligned} \partial _\mu \left( \frac{\partial \mathcal {L}}{\partial (\partial _\mu A_\nu )} \right) - \frac{\partial \mathcal {L}}{\partial A_\nu } = 0 . \end{aligned}$$

Since $\mathcal {L}_{int}$ is independent of $\partial _\mu A_\nu$, using (50),(49), and (47) we find

53 $$\begin{aligned} \frac{\partial \mathcal {L}}{\partial (\partial _\mu A_\nu )}&= \frac{\partial }{\partial (\partial _\mu A_\nu )} \left[ -\frac{1}{4} F_{\alpha \beta } F^{\alpha \beta } \right] , \nonumber \\&= -F^{\mu \nu }. \end{aligned}$$

Next, we evaluate the second term of (52). Since $\mathcal {L}_{free}$ is independent of $A_\nu$, using (51) and (49), we find

54 $$\begin{aligned} \frac{\partial \mathcal {L}}{\partial A_\nu }&= \frac{\kappa }{4} \frac{\partial A^4}{\partial A_\nu }, \nonumber \\&= \kappa A^2 A^\nu . \end{aligned}$$

Using (53) and (54) in (52), we obtain the inhomogeneous nonlinear Maxwell’s equations,

55 $$\begin{aligned} \partial _\mu F^{\mu \nu }&= -\kappa A^2 A^\nu , \nonumber \\&=\sqrt{\mu _0} J^\mu . \end{aligned}$$

### D. Infinitesimal gauge transformation

In this subsection, it is shown that the Lagrangian density is invariant under an infinitesimal gauge transformation up to a total divergence. Consider the infinitesimal gauge transformation,

56 $$\begin{aligned} A_\mu \rightarrow A_\mu ' = A_\mu + \partial _\mu \epsilon . \end{aligned}$$

The gauge invariance of $\mathcal {L}_{free}$ is well known. So, let us consider the second term in (49),

57 $$\begin{aligned} \mathcal {L}_{int} = \frac{\kappa }{4} A^4. \end{aligned}$$

The change in $\mathcal {L}_{int}$ is

58 $$\begin{aligned} \Delta \mathcal {L}_{int}&= \mathcal {L}_{int}' - \mathcal {L}_{int}, \nonumber \\&= \kappa A^2 A^\mu \partial _\mu \epsilon + O(\epsilon ^2). \end{aligned}$$

Ignoring the second or higher order terms in $\epsilon$ and using (45), (58) can be rewritten as

59 $$\begin{aligned} \Delta \mathcal {L}_{int} = -\sqrt{\mu _0} J^\mu \partial _\mu \epsilon . \end{aligned}$$

Consider

60 $$\begin{aligned} \partial _\mu (J^\mu \epsilon ) = (\partial _\mu J^\mu )\epsilon + J^\mu \partial _\mu \epsilon . \end{aligned}$$

Conservation of pseudo-charge yields $\partial _\mu J^\mu =0$ (see (46)) and hence,

61 $$\begin{aligned} \Delta \mathcal {L}_{int} = -\sqrt{\mu _0} \partial _\mu (J^\mu \epsilon ). \end{aligned}$$

The change in the interaction term is a total divergence, i.e., $\Delta \mathcal {L}_{int} = \partial _\mu K^\mu$ where $K^\mu = -\sqrt{\mu _0} J^\mu \epsilon$. Thus, the Lagrangian density is invariant under an infinitesimal gauge transformation up to a total divergence. Since the total divergence does not change the action, it does not change the inhomogeneous nonlinear Maxwell’s equations (55). In other words, the nonlinear Maxwell’s equations ((38)-(41)) are also invariant under the infinitesimal gauge transformation since the homogeneous Maxwell’s equations are the same as those of linear Maxwell’s equations.

### E. Dark soliton solution

Consider the case ${\textbf {A}} = A^{\phi } \boldsymbol{\phi }$ and the rest of the vector EM potential components are zero. Using spherical coordinates, (1) reduces to

62 $$\begin{aligned} \frac{\partial ^2 A^\phi }{\partial r^2} + \frac{4}{r} \frac{\partial A^\phi }{\partial r} + \frac{1}{r^2} \frac{\partial ^2 A^\phi }{\partial \theta ^2} + \frac{3}{r^2} \cot \theta \frac{\partial A^\phi }{\partial \theta } -\frac{1}{c^2} \frac{\partial ^2 A^\phi }{\partial t^2} = \kappa A^2 A^{\phi }, \end{aligned}$$

Consider a spherically symmetric solution with $\partial A^\phi /\partial \theta = 0$. Now (62) becomes

63 $$\begin{aligned} \frac{\partial ^2 A^\phi }{\partial r^2} + \frac{4}{r} \frac{\partial A^\phi }{\partial r} - \frac{1}{c^2} \frac{\partial ^2 A^\phi }{\partial t^2} = \kappa r^2 \sin ^2 \theta (A^{\phi })^3, \end{aligned}$$

To remove the $\theta$ dependence in the nonlinear term on the right-hand side of (63), (63) is integrated over the cross-section to obtain

64 $$\begin{aligned} \frac{\partial ^2 A^\phi }{\partial r^2} + \frac{4}{r} \frac{\partial A^\phi }{\partial r} - \frac{1}{c^2}\frac{\partial ^2 A^\phi }{\partial t^2} = \kappa _{eff} r^2 (A^{\phi })^3, \end{aligned}$$

where

65 $$\begin{aligned} \kappa _{eff}&= \frac{\kappa \int _0^{2 \pi } \int _0^{\pi } \sin ^3 \theta d\theta d\phi }{\int _0^{2 \pi } \int _0^{\pi } \sin \theta d\theta d\phi } , \nonumber \\&= \frac{2 \kappa }{3}. \end{aligned}$$

A similar approach is used in nonlinear optics to obtain an effective cross-section of the fiber^21,22^. Let

66 $$\begin{aligned} A^{\phi } = \frac{1}{r^2} [ \psi (r) \exp (i\omega t) + c.c. ]. \end{aligned}$$

Substituting (66) in (64), we find

67 $$\begin{aligned} \frac{d^2 \psi }{dr^2} + \left[ \frac{\omega ^2}{c^2} - \frac{2 + \kappa _{eff} \psi ^2(r)}{r^2} \right] \psi = 0. \end{aligned}$$

It can be verified that (64) admits a self-similar solution,

68 $$\begin{aligned} A^{\phi } = \frac{f(\omega r/c) }{\sqrt{\kappa _{eff}} r^2} \exp (i\omega t) + c.c. \end{aligned}$$

Comparing (68) and (66), we find $\psi (r) = f(\omega r/c)/\sqrt{\kappa _{eff}}$. Next, the total mean energy of the dark soliton is calculated as follows. The $\phi$-component of the electric field is

69 $$\begin{aligned} E^{\phi } = -\frac{1}{c} \frac{\partial A^\phi }{\partial t} = -\frac{i \omega f(\omega r/c) }{c\sqrt{\kappa _{eff}} r^2} \exp (i\omega t) + c.c., \end{aligned}$$

and the rest of the electric field components are zero. The mean electric energy density is

70 $$\begin{aligned} \frac{1}{2} \langle {\textbf {E}}.{\textbf {E}} \rangle&= \frac{\eta _{\phi \phi } \langle (E^{\phi })^2 \rangle }{2},\nonumber \\&= \frac{\omega ^2 f^2(\omega r/c) \sin ^2\theta }{c^2\kappa _{eff} r^2}. \end{aligned}$$

Integrating (70) over the volume, we find the mean electric energy as

71 $$\begin{aligned} E_e = \frac{\omega K_e}{c \kappa _{eff}}, \end{aligned}$$

where

72 $$\begin{aligned} K_e = \frac{8 \pi }{3} \int _0^{\infty } f^2 (x) dx .\end{aligned}$$

The $\theta$-component of the magnetic field is

73 $$\begin{aligned} B^{\theta } = -\frac{1}{r^2} \frac{ \partial (r^2 A^\phi ) }{\partial r}, \end{aligned}$$

and the rest of the magnetic field components are zero. Using (68) and (73), we find

74 $$\begin{aligned} B^{\theta } = \frac{ \omega f(\omega r/c) }{c\sqrt{\kappa _{eff}} r^2} \exp (i\omega t) + c.c., \end{aligned}$$

where $'$ denotes the differentiation with respect to the argument. The mean magnetic energy density is

75 $$\begin{aligned} \frac{1}{2} \langle {\textbf {B}}.{\textbf {B}} \rangle&= \frac{\eta _{\theta \theta } \langle (B^{\theta })^2 \rangle }{2} ,\nonumber \\&= \frac{[\omega f'(\omega r/c)]^2 }{c^2\kappa _{eff} r^2}. \end{aligned}$$

Integrating (75) over the volume, the mean magnetic energy is

76 $$\begin{aligned} E_m = \frac{\omega K_m}{c \kappa _{eff}}, \end{aligned}$$

where

77 $$\begin{aligned} K_m = 4 \pi \int _0^{\infty } [f'(x)]^2 dx. \end{aligned}$$

Combining (76) and (71), total electromagnetic energy is

78 $$\begin{aligned} E_{em} = \frac{\omega K_{em}}{c\kappa _{eff}}, \end{aligned}$$

where $K_{em} = K_e + K_m$ is a dimensionless constant. The energy of a dark soliton is unbounded since it becomes a sinusoid as $r \rightarrow \infty$. Typically, the energy of a dark soliton is defined as the difference between the total energy and the energy of the continuous wave (CW) background^22^, i.e.,

79 $$\begin{aligned} K_e = \frac{8 \pi }{3} \lim _{R \rightarrow \infty } \int _0^{R} [S^2\sin ^2(x)-f^2 (x) ] dx, \end{aligned}$$

80 $$\begin{aligned} K_m = 4 \pi \lim _{R \rightarrow \infty } \int _0^{R} [S^2\cos ^2(x)-f'(x)]^2 ] dx, \end{aligned}$$

81 $$\begin{aligned} K_{em}' = K_e + K_m , \end{aligned}$$

where *S* is the amplitude of *f*(*x*) as $x \rightarrow \infty$. Note that the renormalization of the dark soliton is not unique. The excess Hamiltonian of the soliton solution over the CW background can also be used for renormalization, yielding a different value of $K_{em}'$. For the parameters of Fig. 3, using (79) and (80), we find $K_{em}' = 1.06\times 10^4$. Now, using (5), (65), and replacing $K_{em}$ in (78) with $K_{em}'$, the mean energy of a dark soliton can be written as

82 $$\begin{aligned} E_{em}'&= \frac{\omega K_{em}'}{c\kappa _{eff}}, \nonumber \\&= M\hbar \omega , \end{aligned}$$

where $$M = 3K_{em}/2 = 1.59\times 10^4$$.

### F. Curve fitting the equilibrium spectrum with the Planck distribution

First, (1) is normalized using

83 $$\begin{aligned} x'^\mu = x^\mu /l_0, \hspace{1in} D^\mu = A^\mu \sqrt{\frac{l_0}{\mathcal {E}_0}} , \end{aligned}$$

where $l_0$ and $\mathcal {E}_0$ are suitably chosen distance and energy scaling factors, respectively. In our units (see SI), the unit of $A^\mu$ is $\sqrt{J/m}$ and hence, $D^\mu$ is dimensionless. Now, (1) may be rewritten in the dimensionless form as

84 $$\begin{aligned} \nabla '^2 D^\mu - \frac{\partial ^2 D^\mu }{\partial t^2} = \kappa ' g_{\alpha \beta }D^{\alpha }D^{\beta } D^{\mu }, \end{aligned}$$

where

85 $$\begin{aligned} t' = x'^0, \end{aligned}$$

86 $$\begin{aligned} \kappa ' = \kappa \mathcal {E}_0 l_0. \end{aligned}$$

The unit of $\kappa$ is 1/(*J*.*m*) and hence, $\kappa '$ is dimensionless. In the absence of the nonlinear term on the right-hand side of (& 84), it admits TE modes and TM modes. Only TE modes are considered here, assuming TM modes are absent ($D^z = D^0 = 0$). The periodic boundary conditions are used, i.e., $D^j (x'^\mu ) = D^j (x'^\mu +L'),\hspace{0.1in} j=x,y$, where $L'=L/l_0$. In the absence of the nonlinear term on the right-hand side of (84), the vector potential components of the TE modes are given by^14^

87 $$\begin{aligned} D^x= D^{x0} \cos (2\pi n_x x'/L') \sin (2\pi n_y y'/L') \sin (2\pi n_z z'/L') \cos (\Omega t'), \nonumber \\ D^y= - \frac{n_x D^{x0}}{n_y} \sin (2\pi n_x x'/L') \cos (2\pi n_y y'/L') \sin (2\pi n_z z'/L') \cos (\Omega t'), \end{aligned}$$

where $n_x,n_y$ and $n_z$ are integers and $\Omega = 2\pi \sqrt{n_x^2 + n_y^2 + n_z^2}/L'$. (84) is solved numerically using the fourth-order Runge-Kutta technique (SI). If $D^x$ and $D^y$ are known at $t'$, they are calculated at each point on the computational grid ($l \Delta x, m \Delta y, n \Delta z$) at $t'+\Delta t'$. The following initial conditions are used,

88 $$\begin{aligned} D^x ({\textbf {r}}', t'=0)= B_{0} \sum _{n_x=1}^{4} \sum _{n_y=1}^4 \sum _{n_z=1}^4 \frac{1}{\Omega }\cos (2\pi n_x x'/L') \sin (2\pi n_y y'/L') \sin (2\pi n_z z'/L'), \nonumber \\ D^y ({\textbf {r}}',t'=0)= - B_{0} \sum _{n_x=1}^{4} \sum _{n_y=1}^4 \sum _{n_z=1}^4 \frac{n_x}{n_y \Omega } \cos (2\pi n_x x'/L') \sin (2\pi n_y y'/L') \sin (2\pi n_z z'/L'), \end{aligned}$$

where ${\textbf {r}}' = (x',y',z')$. To see the resemblance between the blackbody spectrum and the equilibrium spectrum of Fig 2, a curve fitting is done to the simulated spectrum of Fig. 2 with $B_0$ = 0.25, using the Planck distribution. According to Planck’s law, the normalized energy spectral density is

89 $$\begin{aligned} S(f) = \frac{a f^3 }{\exp {(bf})-1}, \end{aligned}$$

where

90 $$\begin{aligned} a = \frac{4 \pi h c}{l_0\mathcal {E}_0}, \end{aligned}$$

91 $$\begin{aligned} b = \frac{h c }{l_0 \kappa _B T}. \end{aligned}$$

In (89) and (90), it is assumed that only one polarization component is present in the cavity since our simulation includes only the TE mode.

Due to the self-similarity, the general solution of (84) can be written as

92 $$\begin{aligned} D^\mu (\mathbf{r'}, t') = \frac{\eta \psi ^\mu (\eta \mathbf{r'},\eta t)}{\sqrt{\kappa '}}. \end{aligned}$$

The Fourier transform of (92) is

93 $$\begin{aligned} \tilde{D}^\mu (\mathbf{r'}, \omega ) = \frac{\eta \tilde{\psi }^\mu (\eta \mathbf{r'},\omega )}{\sqrt{\kappa '}} . \end{aligned}$$

The normalized EM energy spectral density is

94 $$\begin{aligned} S(f)&\propto |\tilde{E}(\omega )|^2 + |\tilde{B}(\omega )|^2, \end{aligned}$$

95 $$\begin{aligned}&\propto \sum _{\mu =1}^2 \frac{\eta ^3 |\tilde{\psi }^\mu (\eta \mathbf{r'},\omega )|^2}{\kappa '}, \end{aligned}$$

96 $$\begin{aligned}&\propto \frac{1}{\kappa '}, \end{aligned}$$

where

97 $$\begin{aligned} |\tilde{E}(\omega )|^2 = |\tilde{E}_x(\omega )|^2 + |\tilde{E}_y(\omega )|^2, \end{aligned}$$

98 $$\begin{aligned} |\tilde{B}(\omega )|^2 = |\tilde{B}_x(\omega )|^2 + |\tilde{B}_y(\omega )|^2 + |\tilde{B}_z(\omega )|^2, \end{aligned}$$

$\tilde{E}_l(\omega ), l=x,y$ and $\tilde{B}_l(\omega ), l=x,y,z$ are the Fourier transforms of electric and magnetic field intensities, respectively. From (96), we see that the energy spectral density *S*(*f*) is inversely proportional to $\kappa '$, and comparing (96) and (89), we find

99 $$\begin{aligned} a = \frac{\alpha }{\kappa '}, \end{aligned}$$

where $\alpha$ is a constant to be determined. From (86), (90) and (99), it follows that

100 $$\begin{aligned} \kappa = \frac{\kappa '}{l_0\mathcal {E}_0} =\frac{\alpha }{4\pi h c} . \end{aligned}$$

The best curve fitting occurs when *b* = 5.8 and *a* = 833.88 (See Fig. 2). Using this value of *a*, and with $\kappa '$ = 0.1 (this is the value used in the numerical simulations), we find that $\alpha = 83.38 \approx 8\pi ^2$ and (100) becomes

101 $$\begin{aligned} \kappa \approx \frac{1}{\hbar c}. \end{aligned}$$

### G. Relationship between Planck’s constant and pseudo-charge

Consider the spherically symmetric and time-independent solution of (11). If we ignore the terms that are dependent on *t*,$\theta$, and $\phi$ in (11), we obtain

102 $$\begin{aligned} \frac{d^2 A^0}{d r^2} + \frac{2}{r} \frac{d A^0}{d r} = \kappa A^2 A^0, \end{aligned}$$

Due to the self-similar property of (102), its general solution can be written as

103 $$\begin{aligned} A^0 = \frac{\eta }{\sqrt{\kappa }} f(\eta r). \end{aligned}$$

As $r\rightarrow \infty$, the nonlinear term on the right-hand side of (102) vanishes and therefore, $f(x) \rightarrow N/x$ as $x \rightarrow \infty$, where *N* is a dimensionless constant determined by the boundary condition at $\infty$. Using (103), as $r\rightarrow \infty$, we have

104 $$\begin{aligned} A^0 \rightarrow \frac{N}{\sqrt{\kappa }r}. \end{aligned}$$

The pseudo-charge is defined as (see (12)).

105 $$\begin{aligned} q_{p} = -\int \sqrt{\epsilon _0}\kappa A^2 A^0 dV. \end{aligned}$$

Using Gauss’s divergence theorem, we have

106 $$\begin{aligned} \frac{q_p}{\sqrt{\epsilon _0}} = \int _V \nabla . \textbf{E} dV = \int _S \textbf{E}. d\textbf{S}, \end{aligned}$$

where *S* can be the surface of a sphere with it radius $R \rightarrow \infty$. Using $\textbf{E} = -\partial A^0/\partial r \textbf{r}$ and using (104), as $R\rightarrow \infty$, we have

107 $$\begin{aligned} \textbf{E} \rightarrow -\frac{N}{\sqrt{\kappa }R^2}\textbf{r}. \end{aligned}$$

Using (107) in the surface integral of (106), we obtain

108 $$\begin{aligned} q_p =-\frac{4\pi \sqrt{\epsilon _0} N}{\sqrt{\kappa }}. \end{aligned}$$

Finally, using (5), we find

109 $$\begin{aligned} q_p^2 = 4\pi \epsilon _0 \hbar c K, \end{aligned}$$

where $$K = 4\pi N^2$$.

## Supplementary Information

The supplementary information is available in Figshare with the title, Supplementary information for the paper “The Connection Between Nonlinear Extension of Maxwell’s Equations, Blackbody Spectrum, Lorentz Force and Quantum Mechanics”, identifier DOI 10.6084/m9.figshare.30226492.

## Supplementary Information

Supplementary Information.

## Data Availability

The data that support the findings of this study are available in Zenodo with the identifier DOI 10.528/zenodo. 16364486.
