# Supplementary material for: The connection between nonlinear extension of Maxwell’s equations, blackbody spectrum, Lorentz force and quantum mechanics
Source: Sci Rep. 2026 Jan 19;16:4269. doi: 10.1038/s41598-025-34478-2 (PMC12858852; doi:10.1038/s41598-025-34478-2)
Supplement: Supplementary file 1 — Supplementary Information. [file 41598_2025_34478_MOESM1_ESM.pdf]

Supplementary Information

Shiva Kumar

Electrical and Computer Engineering, ITBA-314, McMaster University,

1280 Main St. W., Hamilton, ON-L8S 4K1, Canada

The problems of classical electrodynamics can be divided into two classes. (i) The charge and current distributions are known and the resulting electromagnetic (EM) fields are calculated, and (ii) the external EM fields are specified and the motion of charged particles under the influence of EM fields is calculated [1]. When these two problems are combined as in the case of bremsstrahlung, the classical treatment is a two-step process: (i) the motion of charged particles in the external field is determined ignoring the emission of radiation by the charged particles, and then (ii) Maxwell's equations are solved to find the EM fields taking into account the trajectory of the moving charges. This way of handling problems in electrodynamics is of approximate validity since the emitted radiation due to accelerating charges carries off energy and momentum, and so must influence the subsequent motion of charged particles. A correct treatment must include the reaction of radiation on the motion of sources. Although there have been many attempts to include radiation reaction [2–5], according to Jackson [1], a completely satisfactory classical treatment of reactive effects of the radiation does not exist. However, a semiclassical theory in which the field is treated classically and the charged matter is treated quantum mechanically, contain the back-action of the radiation field on the charge [6]. The nonlinear Maxwell's equations proposed here describe the evolution of the field distribution, as well as providing the Lorentz force equation to describe the motion of pseudo-charges.

Planck modeled the walls of the cavity as tiny harmonic oscillators that emit or absorb radiation energy, which can take only discrete values, $E_n = nhf$, where n is an integer, h is Planck's constant, and f is the frequency of radiation [7]. Building on this idea, Einstein postulated that the electromagnetic (EM) radiation consists of discrete packets of energy (now called photons) and the energy of each photon is proportional to the frequency of radiation [8]. Using this idea, he explained the photoelectric effect, which was not possible to explain using Maxwell's equations and Lorentz force equations. In linear systems, the amplitude or energy of a wave packet and its mean frequency are two independent degrees of freedoms. However, in nonlinear optics, certain types of nonlinear waves and solitons possess a property that the change in the mean wavenumber of the wave packet is proportional to its energy. If we replace the space variable with a time variable, this translates as the change in the mean frequency of the wave packet is proportional to its energy. Motivated by this fact, the possibility of introducing a nonlinear term to Maxwell's equations is explored in this paper. It is shown that nonlinear Maxwell's equations admit dark soliton solutions

whose energy is proportional to hf, where f is the mean frequency of the dark soliton. The dark soliton is not localized in space; it is a "hole" in the intensity with a continuous wave background, i.e., it has infinite spatial extent with intensity dropping as $1/r^2$.

In the classical description of an atom that uses linear Maxwell's equations and Lorentz force equations, an orbiting electron radiate energy and spiral into the nucleus. This was a major flaw in the early atomic models and one of the key reasons for the development of the Schrodinger equation and quantum mechanics. In this paper, it is shown that under the slowly varying envelope approximation, the Schrodinger equation can be obtained from nonlinear Maxwell's equations. The similarity between the Helmholtz equation under the paraxial approximation (used in optics) and the Schrodinger equation of quantum mechanics is well known if one replaces the space variable of the Helmholtz equation with the time variable, and the refractive index profile with the potential function. However, this similarity was considered accidental. But with the proposed nonlinear extension of Maxwell's equations, the connection becomes evident.

I. LORENTZ INVARIANCE OF NONLINEAR MAXWELL'S EQUATIONS

It is postulated that EM propagation in a vacuum is described by the nonlinear Maxwell's equations,

$$\nabla^2 A^\mu - \frac{1}{c^2}\frac{\partial^2 A^\mu}{\partial t^2} = \kappa A^2 A^\mu, \tag{1}$$

with the Lorentz gauge condition

$$\partial_\mu A^\mu = 0, \tag{2}$$

where $A^2 = g_{\alpha\beta}A^\alpha A^\beta$, A^μ is the vector potential, $g_{\alpha\beta}$ is Minkowski tensor, κ is the nonlinear coefficient of vacuum, $x^0, x^1, x^2, x^3 = ct, x, y, z$, and ∇^2 is the three-dimensional Laplacian operator. Consider an observer moving with speed v in the z-direction relative to a reference frame in which (1) holds. The moving observer's reference frame is

$$\begin{aligned} z' &= \gamma(z - vt), \\ t' &= \gamma(-vz/c^2 + t), \\ x' &= x, \\ y' &= y, \end{aligned} \tag{3}$$

where $\gamma = 1/\sqrt{1-(v/c)^2}$. The vector potentials transform as

$$\begin{aligned} (A^z)' &= \gamma(A^z - vA^0/c), \\ (A^0)' &= \gamma(-vA^z/c + A^0), \\ (A^x)' &= A^x, \\ (A^y)' &= A^y. \end{aligned} \tag{4}$$

Since the length A^2 is invariant under the Lorentz transformation, we have

$$A^2 = g_{\alpha\beta}A^\alpha A^\beta = g_{\alpha\beta}(A^\alpha)'(A^\beta)'. \tag{5}$$

(4) can be inverted to obtain

$$\begin{aligned} A^z &= \gamma[(A^z)' + v(A^0)'/c], \\ A^0 &= \gamma[v(A^z)'/c + (A^0)'], \\ A^x &= (A^x)', \\ A^y &= (A^y)'. \end{aligned} \tag{6}$$

Since the linear scalar wave equation is invariant under the Lorentz transformation, using (6), we have

$$\left[\nabla^2 - \frac{1}{c^2}\frac{\partial^2}{\partial t^2}\right] A^z = \left[\nabla'^2 - \frac{1}{c^2}\frac{\partial^2}{\partial t'^2}\right] \gamma[(A^z)' + v(A^0)'/c], \tag{7}$$

where ∇'^2 is the Laplacian operator in the primed coordinate system. Using (5) and (6) on the right-hand side of (1) with $\mu = z$, we find

$$\kappa g_{\alpha\beta}A^\alpha A^\beta A^z = \kappa g_{\alpha\beta}(A^\alpha)'(A^\beta)'\gamma[(A^z)' + v(A^0)'/c]. \tag{8}$$

Combining (7) and (8), we have

$$\left[\nabla'^2 - \frac{1}{c^2}\frac{\partial^2}{\partial t'^2}\right] [(A^z)' + v(A^0)'/c] = \kappa g_{\alpha\beta}(A^\alpha)'(A^\beta)'[(A^z)' + v(A^0)'/c]. \tag{9}$$

Similarly, the time component of (1) transforms as

$$\left[\nabla'^2 - \frac{1}{c^2}\frac{\partial^2}{\partial t'^2}\right] [v(A^z)'/c + (A^0)'] = \kappa g_{\alpha\beta}(A^\alpha)'(A^\beta)'[v(A^z)'/c + (A^0)']. \tag{10}$$

Multiplying (10) by v/c and subtracting it from (9), we find that the z-component of the vector potential is invariant:

$$\left[\nabla'^2 - \frac{1}{c^2}\frac{\partial^2}{\partial t'^2}\right] (A^z)' = \kappa g_{\alpha\beta}(A^\alpha)'(A^\beta)'(A^z)'. \tag{11}$$

Similarly, multiplying (9) by v/c and subtracting it from (10), we find that the time component of the vector potential is invariant:

$$\left[\nabla'^2 - \frac{1}{c^2}\frac{\partial^2}{\partial t'^2}\right](A^0)' = \kappa g_{\alpha\beta}(A^\alpha)'(A^\beta)'(A^0)'. \tag{12}$$

Since $A^x = (A^x)'$ and $A^y = (A^y)'$, x- and y- components of the vector potentials are also invariant under Lorentz transformation. Lorentz invariance of the Lorentz gauge condition (2) is well-known [1].

II. UNITS AND DIMENSIONS

The units used in this paper are almost identical to the SI units, with a minor difference. In SI units, conventional linear Maxwell's equations in free space are given by

$$\begin{aligned}
\nabla.\mathbf{E} &= \frac{\rho}{\epsilon_0}, \\
\nabla.\mathbf{B} &= 0, \\
\nabla\times\mathbf{E} &= -\frac{\partial \mathbf{B}}{\partial t}, \\
\nabla\times\mathbf{B} &= \mu_0\mathbf{J} + \mu_0\epsilon_0\frac{\partial \mathbf{E}}{\partial t},
\end{aligned} \tag{13}$$

and EM energy density is

$$u = \frac{1}{2}\left(\epsilon_0 E^2 + \frac{B^2}{\mu_0}\right). \tag{14}$$

We define

$$\mathbf{E}' = \sqrt{\epsilon_0}\mathbf{E}, \qquad \mathbf{B}' = \frac{\mathbf{B}}{\sqrt{\mu_0}}, \tag{15}$$

so that ϵ_0 and μ_0 do not appear explicitly in the expression for energy density with the new definition of electric and magnetic fields, i.e., (14) becomes

$$u = \frac{1}{2}\left(E'^2 + B'^2\right). \tag{16}$$

Since the unit of u in SI units is J/m^3, the unit of E' (or B') becomes $\sqrt{J/m^3}$.

Using (15), (13) becomes

$$\nabla.\mathbf{E}' = \frac{\rho}{\sqrt{\epsilon_0}},$$
$$\nabla.\mathbf{B}' = \mathbf{0},$$
$$\nabla \times \mathbf{E}' = -\frac{1}{c}\frac{\partial \mathbf{B}'}{\partial t},$$
$$\nabla \times \mathbf{B}' = \sqrt{\mu_0}\mathbf{J} + \frac{1}{c}\frac{\partial \mathbf{E}'}{\partial t}, \tag{17}$$

From now on, we will remove the prime on **E** and **B**, i.e., we assume that the unit of E (or B) is $\sqrt{J/m^3}$. Let

$$\mathbf{B} = \nabla \times \mathbf{A}, \tag{18}$$
$$\mathbf{E} = -\nabla\phi - \frac{1}{c}\frac{\partial \mathbf{A}}{\partial t}. \tag{19}$$

From (18) and (19), we see that the unit of **A** or ϕ is $\sqrt{J/m}$ and from (1), it follows that the dimension of κ is $1/(J.m)$.

We define the 4-vector potential as $\overrightarrow{\mathbf{A}} = (A^0, A^1, A^2, A^3)$, and $A^0 = \phi$. Using (18),(19), and Lorentz gauge condition (2), (1) can be recast as

$$\nabla \times \mathbf{B} = \sqrt{\mu_0}\mathbf{J} + \frac{1}{c}\frac{\partial \mathbf{E}}{\partial t}, \tag{20}$$

$$\nabla \times \mathbf{E} = -\frac{1}{c}\frac{\partial \mathbf{B}}{\partial t}, \tag{21}$$

$$\nabla.\mathbf{E} = \frac{\rho_p}{\sqrt{\epsilon_0}}, \tag{22}$$

$$\nabla.\mathbf{B} = 0, \tag{23}$$

where

$$\mathbf{J} = -\frac{\kappa A^2 \mathbf{A}}{\sqrt{\mu_0}}, \tag{24}$$

$$\rho_p = -\kappa\sqrt{\epsilon_0}A^2 A^0, \tag{25}$$

$$A^2 = A^\mu A_\mu. \tag{26}$$

We note that the nonlinear Maxwell's equations (20),(21),(22), and (23) have the same form as the linear Maxwell's equations (17). However, the source terms, ρ_p and **J** in (22) and (20), respectively, depend nonlinearly on the vector potentials, as given by (24) and (25).

III. ENERGY CONSERVATION

Using Maxwell's equations (20) and (21), the rate of change of total energy density is [1]

$$\frac{1}{c}\frac{\partial \mathcal{E}}{\partial t} = \sqrt{\mu_0}\mathbf{E}.\mathbf{J} + \frac{1}{2c}\frac{\partial}{\partial t}[\mathbf{E}.\mathbf{E} + \mathbf{B}.\mathbf{B}]. \tag{27}$$

The first and second terms on the right-hand side of (27) represent the mechanical power density and the electromagnetic power density, respectively.

Assuming that the EM wave propagates in z-direction, the EM modes can be divided into two types: (i) Transverse Electric (TE) modes for which the electric field $E_z = 0$, and (ii) Transverse Magnetic (TM) modes for which the magnetic field $B_z = 0$. Thus, for TE modes, we have $A^z = A^0 = 0$, and for TM modes, $A^x = A^y = 0$. First, let us consider the TE mode. With $\mathbf{A} = A^x\mathbf{x} + A^y\mathbf{y}$ and using (24) and (19), the mechanical power density is

$$\begin{aligned}\sqrt{\mu_0}\mathbf{E}.\mathbf{J} &= \frac{\kappa}{c}A^2\mathbf{A}.\frac{\partial \mathbf{A}}{\partial t}, \\ &= \frac{\kappa}{4c}\frac{\partial A^4}{\partial t}.\end{aligned} \tag{28}$$

Using (28) in (27) and integrating, we find the total energy density, up to an additive constant, as

$$\mathcal{E} = \frac{\kappa A^4}{4} + \frac{1}{2}\mathbf{E}.\mathbf{E} + \frac{1}{2}\mathbf{B}.\mathbf{B}. \tag{29}$$

Thus, the total Hamiltonian, which is the spatially integrated energy density, is a constant over time. Total energy density of the TM mode can be similarly calculated.

IV. INTERACTION OF DARK SOLITONS

Let

$$(A^\phi)_j = \frac{1}{r^2}[\psi_j(r)\exp(i\omega_j t) + c.c.], j = 1, 2 \tag{30}$$

be two dark solitons with frequencies ω_1 and ω_2. Let

$$A^\phi = \sum_{j=1}^{2}(A_\phi)_j. \tag{31}$$

Then, we have

$$(A^{\phi})^3 = \frac{1}{r^6}\{[|\psi_1|^2 + 2|\psi_2|^2]\psi_1 \exp(i\omega_1 t)$$
$$+ [|\psi_2|^2 + 2|\psi_1|^2]\psi_2 \exp(i\omega_2 t)$$
$$+ \psi_1^2\psi_2^\star \exp[i(2\omega_1 - \omega_2)t]$$
$$+ \psi_2^2\psi_1^\star \exp[i(2\omega_2 - \omega_1)t] + c.c.\}. \quad (32)$$

From (32), we see that nonlinear interaction leads to new frequency components at $\Omega_1 = 2\omega_1 - \omega_2$ and $\Omega_2 = 2\omega_2 - \omega_1$ due to four-wave mixing (FWM). Therefore, let

$$A^{\phi} = \sum_{j=1}^{2}(A_{\phi})_j + \sum_{j=1}^{2}\frac{\epsilon_j(r,t)}{r^2}\exp(i\Omega_j t) + c.c., \quad (33)$$

where ϵ_j is the FWM component at the frequency Ω_j, generated due to the interaction between the dark solitons, and it is assumed to be small. Substituting (33) in (1) and ignoring the terms proportional to ϵ_j^2 and higher, we obtain

$$-2i\frac{\Omega_j}{c^2}\frac{\partial\epsilon_j}{\partial t} + \frac{\partial^2\epsilon_j}{\partial r^2} + \left(\frac{\omega^2}{c^2} - \frac{2 + 2\kappa_{eff}(|\psi_j|^2 + |\psi_k|^2)}{r^2}\right)\epsilon = \frac{\kappa_{eff}}{r^2}\psi_j^2\psi_k^\star,$$
$$j = 1,2 \qquad k = 3 - j. \quad (34)$$

The terms on the right-hand side of (34) act as a source for the growth of the FWM components ϵ_j. Let $\omega_1 = m\Omega$ and $\omega_2 = (m+1)\Omega$ where Ω is a small fixed frequency. In this case, $\Omega_1 = 2\omega_1 - \omega_2 = (m-1)\Omega$, and $\Omega_2 = 2\omega_2 - \omega_1 = (m+2)\Omega$. Thus, two dark soliton of frequencies $m\Omega$ and $(m+1)\Omega$ generate FWM components of frequencies $(m-1)\Omega$ and $(m+2)\Omega$.

V. NUMERICAL SIMULATION OF THE NONLINEAR MAXWELL'S EQUATIONS

(85) in Methods may be rewritten as

$$\frac{\partial D^{\mu}}{\partial t'} = H^{\mu},$$
$$\frac{\partial H^{\mu}}{\partial t'} = \nabla'^2 D^{\mu} - \kappa' g_{\alpha\beta}D^{\alpha}D^{\beta}D^{\mu}. \quad (35)$$

Although the Laplacian operator can be realized using the finite difference (FD) technique, the numerical accuracy can be significantly enhanced using the three-dimensional Fourier

transform [9]. If we take the three-dimensional Fourier transform of D^μ, the Laplacian operator in (35) can be realized in the frequency domain since

$$\mathcal{F}\{\nabla'^2 D^\mu, x' \to k_x, y' \to k_y, z' \to k_z\} = -k^2 \tilde{D}^\mu(t', k_x, k_y, k_z), \tag{36}$$

where $\mathcal{F}$ denotes the 3-dimensional Fourier transform, $k^2 = k_x^2 + k_y^2 + k_z^2$ and $\tilde{D}^\mu$ denotes the 3-dimensional Fourier transform of D^μ. A 3-dimensional Fourier transform can be conveniently implemented using the fast Fourier transform (FFT). First, we take the three-dimensional FFT of D^μ to obtain $\tilde{D}^\mu$, and it is multiplied by $-k^2$. After taking the inverse FFT of $-k^2\tilde{D}^\mu$, we obtain the desired $\nabla'^2 D^\mu$. The coupled ordinary differential equations (ODEs) given by (35) are numerically solved using the fourth-order Runge-Kutta technique with the initial conditions $D^\mu(0, x', y, z')$ and $H^\mu(0, x', y, z')$. The field components are discretized, i.e., $X^\mu(t', x', y', z') = X^\mu(q\Delta t, l\Delta x, m\Delta y, n\Delta z), l, m, n \in (1, N), X = D$ or H. We chose $L' = 10$ and $N = 80$. We also used the periodic boundary conditions, i.e.,

$$D^\mu(x' + m_x L', y' + m_y L', z' + m_z L') = D^\mu(x', y', z'), \tag{37}$$

where m_x, m_y and m_z are integers. The field components $D^\mu(q\Delta t, l\Delta x, m\Delta y, n\Delta z)$ are stored for $q \in (1, N_t)$ and an FFT is used to obtain the spectrum, $\tilde{D}^\mu(f, l\Delta x, m\Delta y, n\Delta z)$ for each l, m and n. The energy spectral density is obtained by averaging over the three spatial dimensions. The time window of the FFT is so chosen that the frequency resolution is smaller than the fundamental frequency. We chose $N_t = 600$, Δt=0.0313 and the fundamental frequency $= 1/L' = 0.1$. The frequency resolution, $\Delta f = 1/(N_t \Delta t) = 0.0513$. The time resolution, Δt is so chosen that the highest frequency component of the EM field is much smaller than $1/\Delta t$. We have chosen a computational grid of 80×80×80. It may be possible to remove the fluctuations seen in Fig. 1 using a larger grid. However, it would significantly enhance the memory storage requirements, which our computer cannot currently support.

Fig. 1 shows the high intensity peak near the fundamental mode (i.e., the peak near the zero-frequency), which is absent in the black body spectrum. In (1), a simple nonlinear model is used in which the nonlinear refractive index is proportional to A^2. It may be necessary to refine the model by adding the terms proportional to A^4 or A^6 to make a good agreement between the blackbody spectrum and that shown in Fig. 1. On the other hand, the peak near the fundamental mode may be real, and it is linked to the vacuum fluctuations [6, 10]. The fundamental mode of the cavity has the lowest energy (vacuum state). If only

the discrete part of the spectrum shown in Fig. 1 (i.e., the peak near the zero-frequency corresponding to the fundamental mode) is selected and integrated over frequency, using Wiener-Kinchin theorem, it can be shown that the energy density is proportional to the variance of the vector potential components, which can become very large for intense EM radiation stored in the cavity. In quantum optics, the variance of fluctuations of A^2 in the vacuum state could be infinity, which has been the subject of intense debate for a long time which has not been resolved satisfactorily [6, 10].

According to Planck [7], "In a vacuum bounded by totally reflecting walls any state of radiation may persist. But as soon as an arbitrarily small quantity of matter is introduced into the vacuum, a stationary state of radiation is gradually established. It is therefore possible to change a perfectly arbitrary radiation, which exists at the start in the evacuated cavity with perfectly reflecting walls under consideration, into black radiation by the introduction of a minute particle of carbon.". As mentioned before, the vacuum filled with intense EM radiation is not really a vacuum, and the EM radiation is the substitute for the arbitrarily small amount of matter Planck is referring to. In linear Maxwell's equations, there is no way to introduce the effect of EM intensity on its evolution. However, in nonlinear Maxwell's equations, the influence of the EM radiation on its evolution can be described by a nonlinear term in (1).

VI. COULOMB FORCE

The Coulomb interaction between pseudo-charges can be described by the time component (μ=0) of A^μ while the other components (μ=1,2,3) describe the spin of the pseudo-charge. This subsection focuses only on the time component A^0, and the other components are ignored. In this case, (1) reduces to

$$\nabla^2 A^0 - \frac{1}{c^2}\frac{\partial^2 A^0}{\partial t^2} = -\kappa (A^0)^3. \tag{38}$$

Let

$$A^0(\mathbf{r}, t) = \frac{1}{2}[\rho(\mathbf{r}, t)\exp(-i\omega t) + c.c.], \tag{39}$$

where $\rho(\mathbf{r}, t)$ is the slowly varying envelope. Substituting (39) in (38) and making the slowly varying envelope approximation, we obtain

$$2i\frac{\omega}{c^2}\frac{\partial \rho}{\partial t} + \nabla^2 \rho = -\frac{3\kappa}{4}|\rho|^2\rho. \tag{40}$$

While obtaining (40), we have ignored the third harmonic components, $\exp(\pm i3\omega t)$. Let there be two identical pseudo-charges centered at $z_0/2\vec{z}$ and $-z_0/2\vec{z}$ with the separation of z_0 between them. Let

$$\rho(\mathbf{r},t) = \rho_1(\mathbf{r},t) + \rho_2(\mathbf{r},t), \tag{41}$$

where ρ_1 and ρ_2 correspond to the charge distributions of pseudo-charge 1 and 2, respectively. Substituting (41) in (40), we obtain

$$2i\frac{\omega}{c^2}\frac{\partial \rho_j}{\partial t} + \nabla^2\rho_j = -\frac{3\kappa}{4}[(|\rho_j|^2 + 2|\rho_k|^2)\rho_j + \rho_j^2\rho_k^\star]. \quad j = 1, 2, k = 3 - j, \tag{42}$$

Let us consider the impact of pseudo-charge 2 on pseudo-charge 1. With j =1 in (42), we have the self-phase modulation term $|\rho_1|^2\rho_1$ on the right-hand side, which does not contribute to the interaction between the pseudo-charges. Near the center of pseudo-charge 1, ρ_1 is stronger than ρ_2 and hence, the term $\rho_1^2\rho_2^\star$ is more dominant than $2|\rho_2|^2\rho_1$. In fact, the interaction between optical solitons is given by an expression similar to (42), and it is well known that $\rho_1^2\rho_2^\star$ is responsible for attraction/repulsion between the neighboring solitons [11]. So, we consider only the last term of (42) to describe the interaction between the pseudo-charges. Consider $j = 1$ in (42), i.e.,

$$i\frac{\omega}{c^2}\frac{\partial \rho_1}{\partial t} + \frac{1}{2}\nabla^2\rho_1 = -\frac{3\kappa}{8}\rho_1^2\rho_2^\star. \tag{43}$$

The mean of the z-component of the momentum of the pseudo-charge 1 is (see the subsection VI.A)

$$\langle p_z\rangle = \frac{-i\omega}{c^2}\int \rho_1^\star\frac{\partial \rho_1}{\partial z}dV. \tag{44}$$

Using (44), the rate of change of the mean of the z-component of the momentum can be written as

$$\frac{d\langle p_z\rangle}{dt} = \frac{-i\omega}{c^2}\int\left[\frac{\partial \rho_1^\star}{\partial t}\frac{\partial \rho_1}{\partial z} + \rho_1^\star\frac{\partial^2\rho_1}{\partial z\partial t}\right]dV, \tag{45}$$

$$= \frac{-i\omega}{c^2}\int\left[\frac{\partial \rho_1^\star}{\partial t}\frac{\partial \rho_1}{\partial z} - \frac{\partial \rho_1^\star}{\partial z}\frac{\partial \rho_1}{\partial t}\right]dV. \tag{46}$$

In the second term on the right-hand side of (45), we have used integration by parts to obtain (46). Now, multiply (43) by $\partial\rho_1^\star/\partial z$ and integrate over the volume to obtain

$$i\frac{\omega}{c^2}\int\left[\frac{\partial \rho_1}{\partial t}\frac{\partial \rho_1^\star}{\partial z}\right]dV + \frac{1}{2}\int\nabla^2\rho_1\frac{\partial \rho_1^\star}{\partial z}dV = -\frac{3\kappa}{8}\int\rho_1^2\rho_2^\star\frac{\partial \rho_1^\star}{\partial z}dV. \tag{47}$$

Adding the complex conjugate of (47) to itself, and making use of (46), we find

$$\frac{d\langle p_z\rangle}{dt}+\frac{1}{2}\int\left[\nabla^2\rho_1\frac{\partial\rho_1^\star}{\partial z}+\nabla^2\rho_1^\star\frac{\partial\rho_1}{\partial z}\right]dV=-\frac{3\kappa}{4}Re\left\{\int\rho_1^2\rho_2^\star\frac{\partial\rho_1^\star}{\partial z}dV\right\}. \tag{48}$$

The second term in (48) vanishes after integration by parts, leading to

$$\begin{aligned}\frac{d\langle p_z\rangle}{dt}&=\frac{3\kappa}{4}Re\left\{\int\frac{\partial\rho_1^2\rho_2^\star}{\partial z}\rho_1^\star dV\right\},\\&=\frac{3\kappa}{4}Re\left\{\int\left[\rho_1^2\frac{\partial\rho_2^\star}{\partial z}+2\rho_1\rho_2^\star\frac{\partial\rho_1}{\partial z}\right]\rho_1^\star dV\right\}.\end{aligned} \tag{49}$$

Using

$$\tilde{E}_j=-\frac{\partial\rho_j}{\partial z},\qquad j=1,2, \tag{50}$$

where $\tilde{E}_j$ represents the slowly varying envelope of the z-component of the electric field intensity due to the pseudo-charge j, (49) may be rewritten as

$$\frac{d\langle p_z\rangle}{dt}=-\frac{3\kappa}{4}Re[\langle\rho_1|\rho_1^\star\tilde{E}_2|\rho_1\rangle+2\langle\rho_1|\rho_2\tilde{E}_1^\star|\rho_1\rangle]. \tag{51}$$

A. Mean Momentum of a pseudo-charge

Consider a single pseudo-charge whose field evolution is described by (40). Due to self-similarity, the general solution of (40) may be written as

$$\rho(\mathbf{r},t)=\frac{k}{\sqrt{\kappa}}f(k\mathbf{r},\omega t), \tag{52}$$

where $k=\omega/c$. We assume that f is real. If (52) is a solution of (40), due to the Galilean invariance of (40), it follows that

$$\rho(\mathbf{r},t)=\frac{k}{\sqrt{\kappa}}f(kx,ky,k(z-v_zt),\omega t)\exp[ikv_zz/c-\omega v_z^2t/(2c^2)], \tag{53}$$

is also a solution of (40).

In soliton literature, the mean of the z-component of the momentum of a wave packet is defined as $Im\left\{\int\rho^\star\frac{\partial\rho}{\partial z}dV\right\}$ whether or not it is a soliton [11, 12]. In this subsection, using (53), it is shown that it is proportional to the mean momentum of a pseudo-charge. Consider

$$\frac{\omega}{c^2}Im\left\{\int\rho^\star\frac{\partial\rho}{\partial z}dV\right\}=\frac{\omega k^2}{c^2\kappa}Im\left\{\int f\left[\frac{\partial f}{\partial z}+\left(\frac{ikv_z}{c}\right)f\right]dV\right\}. \tag{54}$$

The first term on the right-hand side vanishes after taking the imaginary part. From the second term and using $\kappa = 1/(\hbar c)$, (54) simplifies to

$$\frac{\omega}{c^2} Im \left\{ \int \rho^\star \frac{\partial \rho}{\partial z} dV \right\} = \frac{v_z \hbar \omega k^3}{c^2} \int f^2(kx, ky, k(z - v_z t), \omega t) dx dy dz,$$
$$= a v_z \frac{\hbar \omega}{c^2}, \quad (55)$$

where a is a dimensionless constant,

$$a = \int f^2(x', y', z', \omega t) dx' dy' dz', \quad (56)$$

with $x' = kx$, $y' = ky$, and $z' = k(z - v_g t)$. If we interpret $\frac{\hbar\omega}{c^2}$ as the effective mass of the pseudo-charge, from (55), we find that $\frac{\omega}{c^2} Im \left\{ \int \rho^\star \frac{\partial \rho}{\partial z} dV \right\}$ is proportional to the z-component of the momentum with the constant of proportionality being a. Either we can normalize f such that a is unity, or the right-hand side of (51) (or (74)) can be divided by a.

Finally, since the momentum is real, (55) may be rewritten as

$$\frac{\omega}{c^2} Im \left\{ \int \rho^\star \frac{\partial \rho}{\partial z} dV \right\} = \frac{\omega}{c^2} \int dV \rho^\star \left(-i \frac{\partial}{\partial z} \right) \rho. \quad (57)$$

VII. LORENTZ FORCE

Consider an observer moving at the speed v_x in the x-direction. To simplify the analysis, we assume that $|A^x| << |v_x A^0|/c$. Let the reference frame of the observer be K' with primed coordinates. Using the Lorentz transformation, we have

$$x' = \gamma(x - v_x t), \qquad t' = \gamma(t - v_x x/c^2), \qquad y' = y, \qquad z' = z, \quad (58)$$

$$(A^x)' = \gamma(A^x - v_x/c A^0) \approx -\gamma v_x A^0/c, \qquad (A^0)' \approx \gamma A^0, \quad (59)$$

$$(A^y)' = A^y, \qquad (A^z)' = A^z. \quad (60)$$

Let $\vec{A'}_{ext} = (A^x)'_{ext} \vec{x}$ be the vector potential due to an external magnetic field, so that

$$(A^x)'_{tot} = (A^x)'_{ext} - \gamma v_x A^0/c, \quad (61)$$

where $(A^x)'_{tot}$ is the total x-component of the vector potential in the K' frame. Let

$$(A^0)' = \frac{1}{2}[\phi^0 \exp(-i\omega' t') + c.c.], \quad (62)$$

$$(A^x)'_{tot} = \frac{1}{2}[\phi^x_{tot} \exp(-i\omega' t') + c.c.], \tag{63}$$

so that

$$\phi^0 = \gamma\rho, \tag{64}$$

$$\begin{aligned}\phi^x_{tot} &= \phi^x_{ext} - \phi^x, \\ &= \phi^x_{ext} - \gamma v_x \rho/c,\end{aligned} \tag{65}$$

where ϕ^x_{ext} is the slowly varying envelope of $(A^x)'_{ext}$, and

$$\phi^x = \gamma v_x \rho/c. \tag{66}$$

Since (1) is Lorentz invariant, we have

$$\left[\nabla'^2 - \frac{1}{c^2}\frac{\partial^2}{\partial t'^2}\right](A^0)' = \kappa(A')^2(A^0)'. \tag{67}$$

where

$$(A')^2 = A^2 = \{[(A^x)']^2 - [(A^0)']^2\}. \tag{68}$$

Using (62) and (63) in (67) and after doing the slowly varying envelope approximation, we obtain

$$2i\frac{\omega'}{c^2}\frac{\partial\phi^0}{\partial t'} + \nabla'^2\phi^0 = -\frac{3\kappa}{4}\left[\left(|\phi^0|^2 - \frac{2}{3}|\phi^x_{tot}|^2\right)\phi^0 + \frac{1}{3}(\phi^x_{tot})^2(\phi^0)^\star\right]. \tag{69}$$

Using (65), the second term on the right-hand side of (69) can be expanded as

$$|\phi^x_{tot}|^2\phi^0 = \{\gamma^2 v_x^2|\rho|^2/c^2 + |\phi^x_{ext}|^2 - \gamma v_x \rho(\phi^x_{ext})^\star/c - \gamma v_x \rho^\star \phi^x_{ext}/c\}\phi^0. \tag{70}$$

The third and fourth terms on the right-hand side of (70) are mainly responsible for the interaction between the pseudo-charge and external magnetic field. First we focus only on the third term. Multiplying (69) by $\partial(\phi^0)^\star/\partial z'$, adding its complex conjugate, and integrating, we find the rate of change of the z'-component of the momentum of the pseudo-charge as

$$\begin{aligned}\frac{d\langle p'_z\rangle}{dt'} &= -\frac{\kappa v_x}{2c} Re\left\{\int \frac{\partial[(\phi^0)^2(\phi^x_{ext})^\star]}{\partial z'}(\phi^0)^\star dV'\right\}, \\ &= -\frac{\kappa v_x}{2c} Re\left\{\int (\phi^0)^2\frac{\partial(\phi^x_{ext})^\star}{\partial z'}(\phi^0)^\star dV' + 2\int \phi^0\frac{\partial\phi^0}{\partial z'}(\phi^x_{ext})^\star(\phi^0)^\star dV'\right\},\end{aligned} \tag{71}$$

where

$$\langle p'_z\rangle = \frac{-i\omega}{c^2}\int (\phi^0)^\star\frac{\partial\phi^0}{\partial z'}dV'. \tag{72}$$

The slowly varying envelopes of the vector potentials can be related to the magnetic field intensity,

$$\tilde{B}^y_{ext} = \frac{\partial \phi^x_{ext}}{\partial z'}, \qquad \tilde{B}^{y'} = \frac{\partial \phi^x}{\partial z'} = \frac{v_x}{c}\frac{\partial \phi^0}{\partial z'}, \tag{73}$$

where $\tilde{B}^y_{ext}$ and $\tilde{B}^{y'}$ are the slowly varying envelopes of the external magnetic field and that due to the pseudo-charge, respectively. Using (73),(64), and (66), (71) may be rewritten as

$$\begin{aligned}\frac{d\langle p'_z\rangle}{dt'} &= -\frac{\kappa}{2c}Re\left\{\int(\phi^0)^2 v_x(\tilde{B}^y_{ext})^\star(\phi^0)^\star dV' + 2c\int \phi^0\tilde{B}^{y'}(\phi^x_{ext})^\star(\phi^0)^\star dV'\right\},\\ &= -\frac{\kappa}{2c}Re[\langle\phi^0|(\phi^0)^\star v_x(\tilde{B}^y_{ext})|\phi^0\rangle + 2c\langle\phi^0|(\tilde{B}^{y'})^\star\phi^x_{ext}|\phi^0\rangle].\end{aligned} \tag{74}$$

The analysis of the fourth term on the right-hand side of (70) is quite similar.

VIII. PHOTOELECTRIC EFFECT

As in Sections III and IV, we ignore the spin components and focus only on A^0. We assume that the stationary states of the pseudo-charge are of the form

$$A^0_j = \frac{1}{2}[\rho_{0j}(\mathbf{r}) + B_j\rho_j(\mathbf{r})\exp(i\omega_j t) + c.c.], \quad j = 1, 2, 3\ldots \tag{75}$$

We consider the interaction between the two states with frequencies ω_h and ω_l with $\omega_h > \omega_l$ mediated through an external electromagnetic field,

$$A^0_{ext} = \frac{1}{2}[\rho_{ext}(\mathbf{r}, t)\exp(i\omega_{ext}t) + c.c.], \tag{76}$$

where ω_{ext} and ρ_{ext} are its frequency and slowly varying envelope, respectively. The total time component of the vector potential is

$$A^0 = A^0_l + A^0_h + A^0_{ext}. \tag{77}$$

Substituting (77) into (38), using the slowly varying envelope approximation, and separating the terms proportional to $\exp(i\omega_l t), \exp(i\omega_h t)$, and $\exp(i\omega_{ext}t)$, we find

$$2i\frac{\omega_l}{c^2}\frac{dB_l}{dt}\rho_l(\mathbf{r}) = \frac{3}{2}\kappa\rho^r_{0l}B_h\rho_h\rho^\star_{ext}\exp(i\Delta\omega t), \tag{78}$$

$$2i\frac{\omega_h}{c^2}\frac{dB_h}{dt}\rho_h(\mathbf{r}) = \frac{3}{2}\rho^r_{0r}B_l\rho_l\rho_{ext}\exp(-i\Delta\omega t), \tag{79}$$

$$2i\frac{\omega_{ext}}{c^2}\frac{\partial\rho_{ext}}{\partial t} - \nabla^2\rho_{ext} = \frac{3\kappa B_h B^\star_l\rho_h\rho^\star_l(\rho^r_{0r} + \rho^r_{0l})\exp(i\Delta\omega t)}{2}, \tag{80}$$

where $\Delta\omega = \omega_h - \omega_l - \omega_{ext}$ is the FWM phase mismatch, and $\rho_{0j}^r = Re[\rho_{0j}], j = l, h$. Multiplying (78) by $\rho_l^\star(\mathbf{r})$,integrating over the volume and using $\kappa = 1/(c\hbar)$, we find

$$i\hbar \frac{dB_l}{dt} = \Gamma_l B_h \exp(i\Delta\omega t), \tag{81}$$

where Γ_l is the overlap integral,

$$\Gamma_l = \frac{3c}{4\omega_l} \frac{\int \rho_{0l}^r \rho_h \rho_{ext}^\star \rho_l^\star d^3r}{\int |\rho_l|^2 d^3r}. \tag{82}$$

Similarly, multiplying (79) by $\rho_h^\star(\mathbf{r})$ and integrating over the volume, we find

$$i\hbar \frac{dB_h}{dt} = \Gamma_h B_l \exp(-i\Delta\omega t), \tag{83}$$

where

$$\Gamma_h = \frac{3c}{4\omega_h} \frac{\int \rho_{0r}^r \rho_l \rho_{ext} \rho_h^\star d^3r}{\int |\rho_h|^2 d^3r}. \tag{84}$$

(81) and (83) resemble the quantum mechanical equations describing the interaction between an electron bound to an atom and the EM field. For example, if the pseudo-charge is in the lower frequency state at $t = 0$, i.e. $B_l(t = 0) = 1$, and $B_h(t = 0) = 0$, ignoring the decay/growth of the EM field and depletion of the lower frequency state, the solution of (83) may be approximated as

$$B_h(t) = 2\Gamma_h \exp(-i\Delta\omega t/2) \frac{\sin(\Delta\omega t/2)}{\hbar\Delta\omega}, \tag{85}$$

which corresponds to Fermi's golden rule.

---

[1] J. Jackson, *Classical Electrodynamics* (John Wiley and Sons, Inc, 1999).

[2] H. Lorentz, *Theory of Electrons* (Dover, New York, 1952).

[3] P. Dirac, Proceedings of the Royal Society of London. Series A. Mathematical and Physical Sciences **167**, 148 (1938).

[4] G. Ford and R. O'Connell, Phys. Lett. A **157**, 217 (1991).

[5] K. Kim and A. Sessler, The equation of motion of an electron on advanced acceleration concepts, Presented at the 8th workshop, Baltimore, MD (1998).

[6] M. Scully and M. Zubairy, *Quantum Optics* (Cambridge University Press, 1997).

[7] M. Planck, *The Theory of Heat Radiation* (Blakiston's Son Co, Philadelphia, 1914).

[8] A. Einstein, Annalen der Physik **322**, 132 (1905).

[9] A. Taflove and S. Hagness, *Computational Electrodynamics* (Artech House, Boston, 2005).

[10] L. Mandel and E. Wolf, *Optical Coherence and Quantum Optics* (Cambridge University Press, 1995).

[11] A. Hasegawa and Y. Kodama, *Solitons in Optical Communications* (Oxford University Press, Oxford, 1995).

[12] A. Hasegawa, S. Kumar, and Y. Kodama, Reduction of collision-induced time jitters in dispersion-managed soliton transmission systems, Opt. Lett. **21**, 39 (1996).
